# *N*-(2-Chloro­phen­yl)-2-methyl­benzamide

**DOI:** 10.1107/S1600536808020229

**Published:** 2008-07-05

**Authors:** B. Thimme Gowda, Sabine Foro, B. P. Sowmya, Hartmut Fuess

**Affiliations:** a Department of Chemistry, Mangalore University, Mangalagangotri 574 199, Mangalore, India; b Institute of Materials Science, Darmstadt University of Technology, Petersenstrasse 23, D-64287 Darmstadt, Germany

## Abstract

In the structure of the title compound (N2CP2MBA), C_14_H_12_ClNO, the conformations of the N—H and C=O bonds are *trans* to each other. Furthermore, the conformation of the N—H bond is *syn* to the *ortho*-chloro group in the aniline ring and the C=O bond is *syn* to the *ortho*-methyl substituent in the benzoyl ring, similar to what is observed in 2-chloro-*N*-(2-chloro­phen­yl)benzamide and 2-methyl-*N*-phenyl­benzamide. The amide group makes almost the same dihedral angles of 41.2 (14) and 42.2 (13)° with the aniline and benzoyl rings, respectively, while the dihedral angle between the benzoyl and aniline rings is only 7.4 (3)°. The mol­ecules in N2CP2MBA are packed into chains through N—H⋯O hydrogen bonds.

## Related literature

For related literature, see: Gowda *et al.* (2003 ▶, 2008 ▶); Gowda, Foro *et al.* (2007 ▶); Gowda, Sowmya *et al.* (2007 ▶).
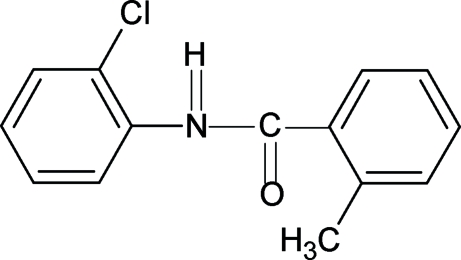


## Experimental

### 

#### Crystal data

C_14_H_12_ClNO
*M* *_r_* = 245.70
Monoclinic, 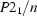

*a* = 4.8881 (4) Å
*b* = 24.318 (2) Å
*c* = 10.0562 (8) Å
β = 90.373 (6)°
*V* = 1195.34 (17) Å^3^
*Z* = 4
Cu *K*α radiation
μ = 2.67 mm^−1^
*T* = 299 (2) K
0.55 × 0.13 × 0.05 mm

#### Data collection

Enraf–Nonius CAD-4 diffractometer
Absorption correction: ψ scan (North *et al.*, 1968 ▶) *T*_min_ = 0.695, *T*_max_ = 0.878
2264 measured reflections
2126 independent reflections
1695 reflections with *I* > 2σ(*I*)
*R*_int_ = 0.050
3 standard reflections frequency: 120 min intensity decay: 1.5%

#### Refinement

*R*[*F* ^2^ > 2σ(*F* ^2^)] = 0.067
*wR*(*F* ^2^) = 0.333
*S* = 1.49
2126 reflections
158 parameters
H atoms treated by a mixture of independent and constrained refinement
Δρ_max_ = 0.58 e Å^−3^
Δρ_min_ = −0.64 e Å^−3^

### 

Data collection: *CAD-4-PC Software* (Enraf–Nonius, 1996 ▶); cell refinement: *CAD-4-PC Software*; data reduction: *REDU4* (Stoe & Cie, 1987 ▶); program(s) used to solve structure: *SHELXS97* (Sheldrick, 2008 ▶); program(s) used to refine structure: *SHELXL97* (Sheldrick, 2008 ▶); molecular graphics: *PLATON* (Spek, 2003 ▶); software used to prepare material for publication: *SHELXL97*.

## Supplementary Material

Crystal structure: contains datablocks I, global. DOI: 10.1107/S1600536808020229/bg2196sup1.cif

Structure factors: contains datablocks I. DOI: 10.1107/S1600536808020229/bg2196Isup2.hkl

Additional supplementary materials:  crystallographic information; 3D view; checkCIF report

## Figures and Tables

**Table 1:** Hydrogen-bond geometry (Å, °)

*D*—H⋯*A*	*D*—H	H⋯*A*	*D*⋯*A*	*D*—H⋯*A*
N1—H1N⋯O1^i^	0.88 (6)	2.03 (6)	2.886 (5)	163 (5)

Symmetry code: (i) 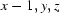.

**Fig. 1.**
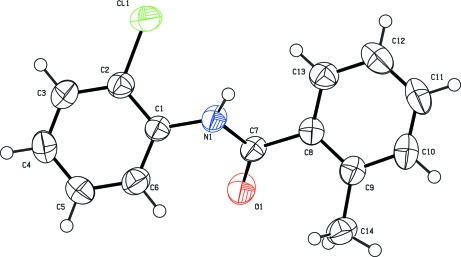
Molecular structure of the title compound, showing the atom labeling. Displacement ellipsoids drawn at the 50% probability level.

**Fig. 2.**
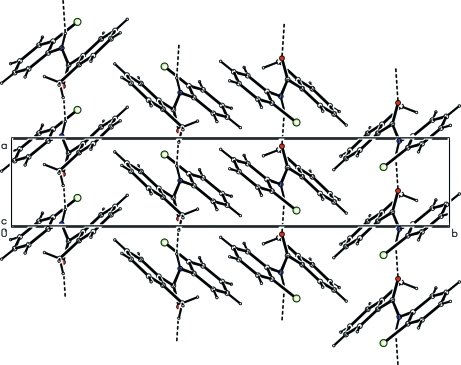
Molecular packing of the title compound with hydrogen bonding shown as dashed lines..

## supplementary crystallographic information

### Comment

As part of a study of the substituent effects on the solid state geometries of benzanilides,in the present work, the structure of 2-methyl-*N*-(2-chlorophenyl)-benzamide (N2CP2MBA) has been determined Gowda *et al.* (2003, 2008). Gowda, Foro *et al.* (2007); Gowda, Sowmya *et al.* (2007). In the structure of N2CP2MBA (Fig. 1), the conformations of the N—H and C═O bonds are *trans* to each other. Further, the conformation of the N—H bond is *syn* to the *ortho*-chloro group in the aniline ring and that of the C═O bond is *syn* to the *ortho*-methyl substituent in the benzoyl ring. These observations are similar to those observed in 2-chloro-*N*-(2-Chlorophenyl)-benzamide (N2CP2CBA) (Gowda *et al.*, 2007*a*) and 2-methyl-*N*-(phenyl)-benzamide (NP2MBA) (Gowda *et al.*, 2008). The bond parameters in N2CP2MBA are similar to those in N2CP2CBA, NP2MBA, *N*-(2-Chlorophenyl)-benzamide and other benzanilides Gowda *et al.* (2003, 2008). Gowda, Foro *et al.* (2007); Gowda, Sowmya *et al.* (2007). The amide group, –NHCO– makes almost the same dihedral angles of 41.2 (14)° and 42.2 (13)° with the aniline and benzoyl rings, respectively, while that between the benzoyl and aniline rings is only 7.4 (3)°. The packing diagram of N2CP2MBA molecules showing the hydrogen bonds N—H···O (Table 1) involved in the formation of molecular chains is shown in Fig. 2.

### Experimental

The title compound was prepared according to the literature method (Gowda *et al.*, 2003). The purity of the compound was checked by determining its melting point. It was characterized by recording its infrared and NMR spectra. Single crystals of the title compound were obtained from an ethanolic solution and used for X-ray diffraction studies at room temperature.

### Refinement

The NH atom was located in difference map and refined with a restrained N—H = 0.88 (6) Å. The other H atoms were positioned with idealized geometry using a riding model with C—H = 0.93–0.96 Å. All H atoms were refined with isotropic displacement parameters (set to 1.2 times of the *U*_eq_ of the parent atom). In spite of the refinement proceeding and converging smoothly, the R2 index did  not decrese from a rather large value (0.33). This might be atributed to poor crystal quality in the available samples.

### Crystal data

**Table:**

C_14_H_12_ClNO	*F*_000_ = 512
*M**_r_* = 245.70	*D*_x_ = 1.365 Mg m^−^^3^
Monoclinic, *P*2_1_/*n*	Cu *K*α radiation λ = 1.54180 Å
Hall symbol: -P 2yn	Cell parameters from 25 reflections
*a* = 4.8881 (4) Å	θ = 7.3–21.6º
*b* = 24.318 (2) Å	µ = 2.67 mm^−^^1^
*c* = 10.0562 (8) Å	*T* = 299 (2) K
β = 90.373 (6)º	Rod, colourless
*V* = 1195.34 (17) Å^3^	0.55 × 0.13 × 0.05 mm
*Z* = 4	

### Data collection

**Table:**

Enraf–Nonius CAD-4 diffractometer	*R*_int_ = 0.050
Radiation source: fine-focus sealed tube	θ_max_ = 66.9º
Monochromator: graphite	θ_min_ = 3.6º
*T* = 299(2) K	*h* = −5→5
ω/2θ scans	*k* = −29→0
Absorption correction: ψ scan(North *et al.*, 1968)	*l* = −12→1
*T*_min_ = 0.695, *T*_max_ = 0.878	3 standard reflections
2264 measured reflections	every 120 min
2126 independent reflections	intensity decay: 1.5%
1695 reflections with *I* > 2σ(*I*)	

### Refinement

**Table:**

Refinement on *F*^2^	Secondary atom site location: difference Fourier map
Least-squares matrix: full	Hydrogen site location: inferred from neighbouring sites
*R*[*F*^2^ > 2σ(*F*^2^)] = 0.067	H atoms treated by a mixture of independent and constrained refinement
*wR*(*F*^2^) = 0.333	*w* = 1/[σ^2^(*F*_o_^2^) + (0.2*P*)^2^] where *P* = (*F*_o_^2^ + 2*F*_c_^2^)/3
*S* = 1.49	(Δ/σ)_max_ < 0.001
2126 reflections	Δρ_max_ = 0.59 e Å^−^^3^
158 parameters	Δρ_min_ = −0.64 e Å^−^^3^
Primary atom site location: structure-invariant direct methods	Extinction correction: none

### Special details

**Table:**

Geometry. All e.s.d.'s (except the e.s.d. in the dihedral angle between two l.s. planes) are estimated using the full covariance matrix. The cell e.s.d.'s are taken into account individually in the estimation of e.s.d.'s in distances, angles and torsion angles; correlations between e.s.d.'s in cell parameters are only used when they are defined by crystal symmetry. An approximate (isotropic) treatment of cell e.s.d.'s is used for estimating e.s.d.'s involving l.s. planes.
Refinement. Refinement of *F*^2^ against ALL reflections. The weighted *R*-factor *wR* and goodness of fit *S* are based on *F*^2^, conventional *R*-factors *R* are based on *F*, with *F* set to zero for negative *F*^2^. The threshold expression of *F*^2^ > σ(*F*^2^) is used only for calculating *R*-factors(gt) *etc*. and is not relevant to the choice of reflections for refinement. *R*-factors based on *F*^2^ are statistically about twice as large as those based on *F*, and *R*- factors based on ALL data will be even larger.

### Fractional atomic coordinates and isotropic or equivalent isotropic displacement parameters (Å^2^)

**Table:**

	*x*	*y*	*z*	*U*_iso_*/*U*_eq_	
C1	0.5268 (8)	0.58356 (18)	0.9109 (4)	0.0391 (10)	
C2	0.3925 (9)	0.59484 (18)	0.7921 (5)	0.0435 (10)	
C3	0.4354 (12)	0.5627 (2)	0.6800 (5)	0.0561 (13)	
H3	0.3425	0.5707	0.6014	0.067*	
C4	0.6138 (13)	0.5193 (2)	0.6847 (5)	0.0623 (15)	
H4	0.6438	0.4981	0.6092	0.075*	
C5	0.7482 (11)	0.5073 (2)	0.8016 (5)	0.0576 (13)	
H5	0.8674	0.4776	0.8058	0.069*	
C6	0.7067 (10)	0.5393 (2)	0.9124 (5)	0.0514 (12)	
H6	0.8014	0.5311	0.9904	0.062*	
C7	0.6651 (8)	0.63040 (19)	1.1158 (4)	0.0392 (10)	
C8	0.5627 (8)	0.66363 (17)	1.2305 (4)	0.0377 (10)	
C9	0.6654 (10)	0.65413 (18)	1.3586 (5)	0.0443 (11)	
C10	0.5648 (11)	0.6868 (2)	1.4611 (5)	0.0554 (13)	
H10	0.6293	0.6812	1.5473	0.067*	
C11	0.3720 (12)	0.7272 (2)	1.4380 (6)	0.0632 (15)	
H11	0.3062	0.7481	1.5083	0.076*	
C12	0.2767 (11)	0.7365 (2)	1.3106 (6)	0.0608 (14)	
H12	0.1495	0.7642	1.2945	0.073*	
C13	0.3710 (10)	0.7046 (2)	1.2075 (5)	0.0493 (11)	
H13	0.3054	0.7106	1.1216	0.059*	
C14	0.8698 (11)	0.6100 (2)	1.3901 (5)	0.0549 (13)	
H14A	1.0390	0.6179	1.3461	0.066*	
H14B	0.8010	0.5751	1.3599	0.066*	
H14C	0.9004	0.6086	1.4844	0.066*	
N1	0.4730 (7)	0.61465 (17)	1.0252 (4)	0.0439 (9)	
H1N	0.306 (13)	0.623 (2)	1.050 (5)	0.053*	
O1	0.9068 (6)	0.61743 (16)	1.1044 (3)	0.0565 (10)	
Cl1	0.1723 (3)	0.65063 (6)	0.78357 (13)	0.0601 (6)	

### Atomic displacement parameters (Å^2^)

**Table:**

	*U*^11^	*U*^22^	*U*^33^	*U*^12^	*U*^13^	*U*^23^
C1	0.027 (2)	0.052 (2)	0.038 (2)	0.0007 (16)	0.0020 (16)	−0.0012 (17)
C2	0.037 (2)	0.050 (2)	0.043 (2)	0.0006 (18)	−0.0003 (18)	0.0006 (19)
C3	0.057 (3)	0.067 (3)	0.044 (2)	0.005 (2)	−0.011 (2)	−0.004 (2)
C4	0.067 (3)	0.070 (3)	0.050 (3)	0.011 (3)	0.001 (2)	−0.019 (2)
C5	0.058 (3)	0.058 (3)	0.057 (3)	0.016 (2)	0.003 (2)	−0.005 (2)
C6	0.045 (3)	0.065 (3)	0.044 (2)	0.005 (2)	−0.0031 (19)	0.001 (2)
C7	0.025 (2)	0.058 (3)	0.035 (2)	−0.0011 (17)	0.0025 (16)	0.0022 (18)
C8	0.0257 (19)	0.046 (2)	0.041 (2)	−0.0081 (16)	0.0057 (16)	−0.0031 (18)
C9	0.040 (2)	0.053 (3)	0.041 (2)	−0.0059 (18)	0.0039 (19)	−0.0012 (18)
C10	0.058 (3)	0.065 (3)	0.044 (2)	−0.014 (2)	0.004 (2)	−0.014 (2)
C11	0.058 (3)	0.064 (3)	0.067 (3)	−0.001 (2)	0.014 (3)	−0.028 (3)
C12	0.048 (3)	0.048 (3)	0.086 (4)	0.001 (2)	0.005 (3)	−0.015 (2)
C13	0.039 (2)	0.057 (3)	0.052 (3)	0.0017 (19)	0.002 (2)	0.001 (2)
C14	0.044 (3)	0.072 (3)	0.048 (3)	0.006 (2)	−0.003 (2)	0.007 (2)
N1	0.0276 (18)	0.066 (2)	0.0378 (19)	0.0020 (16)	−0.0001 (15)	−0.0057 (17)
O1	0.0251 (16)	0.092 (3)	0.0523 (19)	0.0015 (16)	0.0009 (13)	−0.0153 (18)
Cl1	0.0564 (9)	0.0687 (10)	0.0551 (9)	0.0175 (5)	−0.0062 (6)	0.0061 (5)

### Geometric parameters (Å, °)

**Table:**

C1—C2	1.387 (6)	C8—C13	1.386 (7)
C1—C6	1.390 (7)	C8—C9	1.399 (6)
C1—N1	1.402 (5)	C9—C10	1.394 (6)
C2—C3	1.388 (7)	C9—C14	1.499 (7)
C2—Cl1	1.734 (5)	C10—C11	1.380 (8)
C3—C4	1.369 (7)	C10—H10	0.9300
C3—H3	0.9300	C11—C12	1.379 (8)
C4—C5	1.375 (8)	C11—H11	0.9300
C4—H4	0.9300	C12—C13	1.377 (7)
C5—C6	1.374 (7)	C12—H12	0.9300
C5—H5	0.9300	C13—H13	0.9300
C6—H6	0.9300	C14—H14A	0.9600
C7—O1	1.229 (5)	C14—H14B	0.9600
C7—N1	1.359 (5)	C14—H14C	0.9600
C7—C8	1.497 (6)	N1—H1N	0.88 (6)
			
C2—C1—C6	117.3 (4)	C10—C9—C8	117.4 (5)
C2—C1—N1	120.6 (4)	C10—C9—C14	119.3 (4)
C6—C1—N1	122.1 (4)	C8—C9—C14	123.2 (4)
C1—C2—C3	121.0 (4)	C11—C10—C9	121.7 (5)
C1—C2—Cl1	119.2 (3)	C11—C10—H10	119.2
C3—C2—Cl1	119.8 (4)	C9—C10—H10	119.2
C4—C3—C2	120.4 (5)	C12—C11—C10	120.0 (5)
C4—C3—H3	119.8	C12—C11—H11	120.0
C2—C3—H3	119.8	C10—C11—H11	120.0
C3—C4—C5	119.6 (5)	C13—C12—C11	119.6 (5)
C3—C4—H4	120.2	C13—C12—H12	120.2
C5—C4—H4	120.2	C11—C12—H12	120.2
C6—C5—C4	120.1 (5)	C12—C13—C8	120.6 (5)
C6—C5—H5	120.0	C12—C13—H13	119.7
C4—C5—H5	120.0	C8—C13—H13	119.7
C5—C6—C1	121.7 (4)	C9—C14—H14A	109.5
C5—C6—H6	119.1	C9—C14—H14B	109.5
C1—C6—H6	119.1	H14A—C14—H14B	109.5
O1—C7—N1	121.7 (4)	C9—C14—H14C	109.5
O1—C7—C8	122.5 (4)	H14A—C14—H14C	109.5
N1—C7—C8	115.8 (3)	H14B—C14—H14C	109.5
C13—C8—C9	120.7 (4)	C7—N1—C1	124.7 (4)
C13—C8—C7	119.2 (4)	C7—N1—H1N	113 (3)
C9—C8—C7	120.0 (4)	C1—N1—H1N	122 (3)
			
C6—C1—C2—C3	0.5 (7)	C13—C8—C9—C10	0.8 (6)
N1—C1—C2—C3	−177.1 (4)	C7—C8—C9—C10	179.2 (4)
C6—C1—C2—Cl1	−178.4 (3)	C13—C8—C9—C14	179.1 (4)
N1—C1—C2—Cl1	3.9 (6)	C7—C8—C9—C14	−2.5 (6)
C1—C2—C3—C4	−0.5 (8)	C8—C9—C10—C11	−0.2 (7)
Cl1—C2—C3—C4	178.5 (4)	C14—C9—C10—C11	−178.6 (5)
C2—C3—C4—C5	0.7 (9)	C9—C10—C11—C12	−0.9 (8)
C3—C4—C5—C6	−1.0 (9)	C10—C11—C12—C13	1.3 (9)
C4—C5—C6—C1	1.1 (8)	C11—C12—C13—C8	−0.7 (8)
C2—C1—C6—C5	−0.8 (7)	C9—C8—C13—C12	−0.4 (7)
N1—C1—C6—C5	176.8 (5)	C7—C8—C13—C12	−178.8 (4)
O1—C7—C8—C13	139.0 (5)	O1—C7—N1—C1	−1.0 (7)
N1—C7—C8—C13	−41.8 (6)	C8—C7—N1—C1	179.8 (4)
O1—C7—C8—C9	−39.4 (6)	C2—C1—N1—C7	−141.4 (5)
N1—C7—C8—C9	139.8 (4)	C6—C1—N1—C7	41.1 (7)

### Hydrogen-bond geometry (Å, °)

**Table:**

*D*—H···*A*	*D*—H	H···*A*	*D*···*A*	*D*—H···*A*
N1—H1N···O1^i^	0.88 (6)	2.03 (6)	2.886 (5)	163 (5)

Symmetry codes: (i) *x*−1, *y*, *z*.

## References

[bb1] Enraf–Nonius (1996). *CAD-4-PC Software* Enraf–Nonius, Delft, The Netherlands.

[bb2] Gowda, B. T., Foro, S., Sowmya, B. P. & Fuess, H. (2007). *Acta Cryst.* E**63**, o3789.

[bb3] Gowda, B. T., Foro, S., Sowmya, B. P. & Fuess, H. (2008). *Acta Cryst.* E**64**, o383. 10.1107/S1600536807068821 PMC2960230 21201413

[bb4] Gowda, B. T., Jyothi, K., Paulus, H. & Fuess, H. (2003). *Z. Naturforsch. Teil A*, **58**, 225–230.

[bb5] Gowda, B. T., Sowmya, B. P., Kožíšek, J., Tokarčík, M. & Fuess, H. (2007). *Acta Cryst.* E**63**, o2906. 10.1107/S1600536807066937 PMC2915381 21200902

[bb6] North, A. C. T., Phillips, D. C. & Mathews, F. S. (1968). *Acta Cryst.* A**24**, 351–359.

[bb7] Sheldrick, G. M. (2008). *Acta Cryst.* A**64**, 112–122. 10.1107/S0108767307043930 18156677

[bb8] Spek, A. L. (2003). *J. Appl. Cryst.* **36**, 7–13.

[bb9] Stoe & Cie (1987). *REDU4* Stoe & Cie, Darmstadt, Germany.

